# Light modulated cnidocyte discharge predates the origins of eyes in Cnidaria

**DOI:** 10.1002/ece3.7280

**Published:** 2021-03-17

**Authors:** Natasha Picciani, Jamie R. Kerlin, Katia Jindrich, Nicholai M. Hensley, David A. Gold, Todd H. Oakley

**Affiliations:** ^1^ Department of Ecology, Evolution and Marine Biology University of California at Santa Barbara Santa Barbara CA USA; ^2^ School of Biosciences Cardiff University Cardiff UK; ^3^ Department of Earth and Planetary Sciences University of California at Davis Davis CA USA; ^4^ Present address: Department of Ecology and Evolutionary Biology Yale University New Haven CT USA; ^5^ Present address: Department of Biology California State University Northridge CA USA

**Keywords:** light sensing, nematocysts, ocelli, photoreception, photosensitivity

## Abstract

Complex biological traits often originate by integrating previously separate parts, but the organismal functions of these precursors are challenging to infer. If we can understand the ancestral functions of these precursors, it could help explain how they persisted and how they facilitated the origins of complex traits. Animal eyes are some of the best studied complex traits, and they include many parts, such as opsin‐based photoreceptor cells, pigment cells, and lens cells. Eye evolution is understood through conceptual models that argue these parts gradually came together to support increasingly sophisticated visual functions. Despite the well‐accepted logic of these conceptual models, explicit comparative studies to identify organismal functions of eye precursors are lacking. Here, we investigate how precursors functioned before they became part of eyes in Cnidaria, a group formed by sea anemones, corals, and jellyfish. Specifically, we test whether ancestral photoreceptor cells regulated the discharge of cnidocytes, the expensive single‐use cells with various functions including prey capture, locomotion, and protection. Similar to a previous study of *Hydra*, we show an additional four distantly related cnidarian groups discharge significantly more cnidocytes when exposed to dim blue light compared with bright blue light. Our comparative analyses support the hypothesis that the cnidarian ancestor was capable of modulating cnidocyte discharge with light, which we speculate uses an opsin‐based phototransduction pathway homologous to that previously described in *Hydra*. Although eye precursors might have had other functions like regulating timing of spawning, our findings are consistent with the hypothesis that photoreceptor cells which mediate cnidocyte discharge predated eyes, perhaps facilitating the prolific origination of eyes in Cnidaria.

## Graphical abstract

Complex biological traits like eyes often evolve by combining previously separate parts that originally served other organismal functions. Here, we investigate eye evolution in Cnidaria by testing whether an ancient role for light sensitivity was to modulate cnidocyte discharge, even before eyes originated. By advancing a possible ancient role for cnidarian photoreceptor cells, our study helps to dissect the functional drivers that can elaborate morphological complexity.

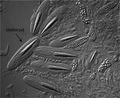

## 1 INTRODUCTION

Complex biological traits often evolve by combining previously separate parts, which we herein term “precursors,” that originally served other organismal functions. Understanding ancestral functions of precursors will help us understand whether and how they were conserved over time, ultimately informing how complex traits originate. An attractive system for exploring the ancestral functions of precursors is animal eyes, which are complex organs composed of modules with known functions, including opsin‐based photoreceptors, pigments, and often lens cells (Oakley & Speiser, 2015). These modules also function outside of eyes, yet only when combined do they facilitate the complex visual tasks that eyes can do. According to a functional model, modules gradually accrued during eye evolution, sequentially adding photoreceptors, pigments, and lenses to support the acquisition of increasingly advanced visual tasks (Nilsson, 2013). The modules did not evolve de novo within eyes but probably were recruited from elsewhere, while also serving functions outside of eyes (Swafford & Oakley, 2019). As such, understanding the functions of precursor modules that would later join forces and become eyes is particularly important for understanding eye origins.

Photoreceptor cells are a logical starting point for understanding eye origins because they are the keystone module of animal eyes. When they are outside of eyes, photoreceptor cells are called extraocular, lack a visual function, and simply sense the ambient intensity of light (Ramirez et al., 2011). Still, they provide nondirectional information on light levels that is useful to organisms for many sensory tasks, including shadow responses, circadian and seasonal entrainment, depth gauges, and other organismal functions (Nilsson, 2009). From the perspective of the functional model of eye evolution, extraocular photoreceptors predated their incorporation into eyes by functioning as simple light gauges for nondirectional photoreception (Nilsson, 2013). Although generally associated with nondirectional photoreception, the organismal‐level functions of eye precursors often go untested.

We propose cnidarians (sea anemones, corals, and jellyfish) are a particularly interesting system for examining possible early functions of eye precursors. Cnidarians convergently evolved eyes of many types in lineages with a jellyfish stage, including lensed eyes with crystallins in box jellyfish (Miranda & Collins, 2019; Picciani et al., 2018). At the same time, ancestral cnidarians lacked eyes altogether but possessed opsin proteins that may have been multimodal (Leung & Montell, 2017) and were probably capable of sensing light (Picciani et al. 2018). Therefore, any functions relying on nondirectional light sensing in the cnidarian ancestor may represent an early role of eye precursors. Nondirectional light sensing in Cnidaria is associated with various sensory tasks, including larval settlement and synchronized mass spawning in corals (Boch et al., 2011; Mason et al., 2012), vertical migration and spawning in jellyfish (Miller, 1979; Quiroga Artigas et al., 2018; Schuyler & Sullivan, 1997), tentacle expansion and retraction in corals and sea anemones (Gorbunov & Falkowski, 2002; Sawyer et al., 1994), and cnidocyte discharge in *Hydra* polyps (Plachetzki et al., 2012). Among these light responses, so far we know that at least two of them are mediated by opsins: light‐induced spawning in the hydrozoan jellyfish *Clytia* (Quiroga Artigas et al., 2018) and light modulation of cnidocyte discharge in *Hydra* (Plachetzki et al., 2012). In the jellyfish *Clytia*, a gonad‐specific opsin of the xenopsin type (opsin9) controls secretion of a neuropeptide that causes oocyte maturation (Quiroga Artigas et al., 2018). Blue/cyan light induces the highest levels of oocyte maturation followed by gamete release, both of which fail to occur in genetically modified gonads that lack *opsin9*. In turn, another xenopsin (HmOps2) expressed in photosensory cells in the tentacles of *Hydra* polyps may modulate the discharge of neighboring stinging cells, the cnidocytes, in response to different intensities of blue light (Plachetzki et al., 2012). Here, the evidence for opsin is not via a knockout experiment, relying instead on a pharmacological agent that targeted a co‐expressed ion channel known to be involved in opsin‐based phototransduction.

**FIGURE 1:**
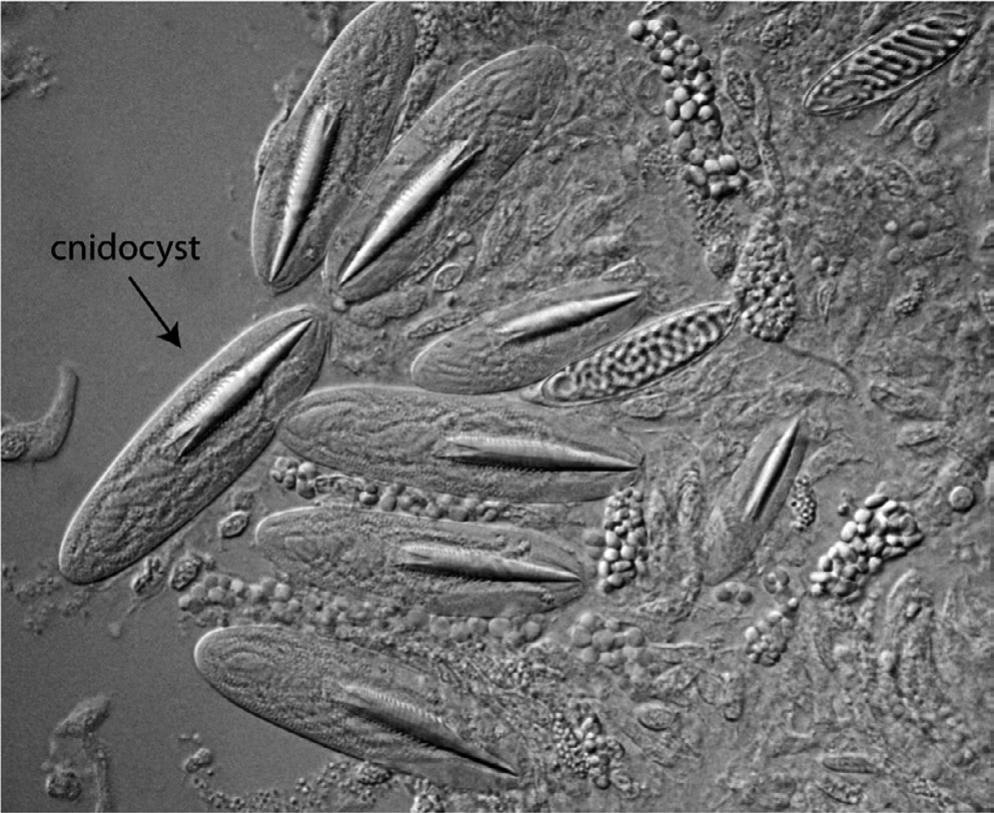
Undischarged cnidocysts from an anthozoan polyp

Because cnidocytes were clearly present in ancestral cnidarians and benefit from strong sensory regulation, we hypothesize modulation of cnidocyte discharge by light was an ancestral function in cnidarians. A cnidocyte is a powerful weapon that produces a ballistic organelle, the cnidocyst, which is discharged upon proper cues (Figure 1; Kass‐Simon et al., 2002). The cnidocyst itself is a capsule, very often containing toxins, with a harpoon‐like tubule that releases its contents after the explosive firing. Cnidocytes are strongly regulated, because they are single‐use and energetically costly to replace (Anderson & Bouchard, 2009). Therefore, to maximize efficient use, multiple sensory modalities, including chemosensation, mechanosensation, and photosensation, regulate cnidocyte discharge, with cnidocytes in the tentacles being highly regulated for efficient prey capture (Anderson & Bouchard, 2009). Assuming sensory regulation was always important for cnidocytes, then both function (regulation) and structure (cnidocyte) may date to the origin of cnidarians. In this study, we investigate whether this nonvisual light response occurs in distantly related groups of Cnidaria other than *Hydra*. Using well‐established cnidocyte capture assays and phylogenetics, we test whether the intensity of blue light also affects the discharge of cnidocytes in four other eyeless species and whether this light response is likely to date to the cnidarian ancestor. Future studies could directly assess the molecular basis of the light modulated cnidocyte discharge. Our study brings into focus the early functional history of light responses in Cnidaria and how ancient sensory tasks may have facilitated eye origins by sustaining simple roles for extraocular photoreceptor cells.

## 2 MATERIALS AND METHODS

### 2.1 Taxon sampling

We tested how light conditions affect cnidocyte capture in four distantly related species, which represent four orders (Corallimorpharia, Actiniaria, Pennatulacea, Semaeostomeae), three subclasses (Hexacorallia, Octocorallia, Discomedusae), and two classes (Anthozoa, Scyphozoa). Most of these species occur in the coast of California and can be cultured over long periods of time, which helps with performing cnidocyte capture assays. We purposely chose distantly related species to span the breadth of Cnidaria, and we acknowledge that future studies that test more species and life stages could improve understanding of the evolution of cnidocyte firing.

### 2.2 Animal cultures

We cultured polyps of the sea anemone *Diadumene lineata* (Verrill, 1869) [=*Haliplanella luciae*] (Actiniaria, Hexacorallia) and the scyphozoan *Aurelia aurita* (Linnaeus, 1758) ("species 1" strain, Semaeostomeae, Discomedusae) in natural seawater at room temperature (22°C ± 1°C) under a 12:12 hr photoperiod with an artificial white fluorescent light. We also cultured specimens of the corallimorph *Corynactis californica* Carlgren, 1936 (Corallimorpharia, Hexacorallia), collected from oil platforms off Santa Barbara, California (USA) on 18 February 2015 and colonies of *Renilla koellikeri* Pfeffer, 1886 (Pennatulacea, Octocorallia), collected in the Santa Barbara Channel on 10 June 2015, in a seawater open system (16°C ± 2°C) with a 12:12 hr photoperiod. Animals were fed 3‐day‐old Selcon®‐enriched *Artemia* nauplii (San Francisco Strain Brine Shrimp Eggs) on a daily basis. We performed all experiments with animals starved for 24 hr.

### 2.3 Cnidocyte assays

Because the polyp is widely accepted to be the ancestral stage among cnidarians, while the pelagic jellyfish evolved later in Medusozoa (Collins, 2002; Collins et al., 2006; Kayal et al., 2018), we focused our experiments on the polyp stage for inferring the ancestral state of the cnidocyte response to light in the cnidarian ancestor. All of our study species produce eyeless polyps, and only *Aurelia* produces a pelagic jellyfish (which possess simple eyes). Additionally, there are three types of cnidocytes (spirocytes, ptychocytes, and nematocytes) among anthozoans, but only the nematocytes are widely distributed across cnidarians. As such, when we refer to cnidocytes throughout the text, we are specifically referring to nematocytes.

Cnidocyte capture assays followed the method described in Watson and Hessinger (1989). After double‐coating fishing line with 20% (w/v) gelatin preheated to ~70°C, 2 cm‐long monofilament fishing line probes (Essentials South Bend®) were left to dry for ~20 min and then used for contacting one tentacle of each individual. We exposed healthy individuals to one of two different light intensities (dim light, 0.1 W/cm^2^; bright light, 2.8 W/cm^2^) from a blue LED (SuperBright LEDs) light source with a spectral peak at 470 nm for approximately two (*Aurelia*, *N* = 33), three (*Corynactis*, *N* = 30; *Renilla*, bright light, *N* = 39; dim light, *N* = 27; maintained at ~ 16°C in a cold chamber during experimentation), or four hours (*Diadumene*, dim light, *N* = 40; bright light, *N* = 33). Because polyps took different amounts of time to relax after being moved into the experimental setup, they were exposed for varying amounts of time. Light intensity was measured using the Jaz spectrometer (Ocean Optics). Gelatin‐coated probes were mounted in 100% glycerol, and discharged nematocysts were counted at 400× or 600× magnification of an Olympus BX61 microscope. We counted nematocysts by searching the whole length and width of the probe (one probe per individual) with proper focal adjustments. Probes were discarded whenever counting could not be done by the lack of a focal point or agglomeration of nematocysts.

### 2.4 Phylogenetic analysis

We used a maximum likelihood approach to infer the ancestral states (light modulated cnidocyte discharge, present or absent) on the time‐calibrated phylogeny from Picciani et al. (2018). We used R 4.0.2 and the function rayDISC from the R package corHMM v1.22 (Beaulieu et al., 2013) to estimate the marginal likelihoods of internal nodes with symmetrical rates model since the asymmetrical one was not significantly better and could lead to overparameterization (likelihood ratio test; chi‐square test; *df* = 1; *p* = 0.1). Because genetic data are scarce for *Renilla,* this species is missing from the phylogeny, and thus, we scored another pennatulacean (*Umbellula*) in our tree as a surrogate taxa. Additionally, because outgroups lack cnidocytes altogether, we used a root prior to fix the root state as absent.

### 2.5 Statistical analysis and accessibility

We analyzed counts of nematocysts captured in the gelatin probes using R 3.6.1. For every species, data were non‐normal (Shapiro–Wilk test, *p* < 0.001; except for *Diadumene,* which had data from treatment with dim light following a normal distribution) and frequency distributions were highly skewed though they had roughly the same shape. Given that, we used the Wilcoxon rank‐sum test to compare sample means of each light treatment, assuming a significance level (α) of 0.05. Data for two species, *Renilla* and *Corynactis*, possessed many trials when no cnidocytes fired. To account for such heavily skewed data, we also used a zero‐inflated negative binomial regression (R package “pscl”) to test whether experimental condition (bright vs. dim light) and species (the four tested, mentioned above) could explain variation in cnidocyte firing counts beyond a statistically distinct process generating the accumulation zeros in our data.

## 3 RESULTS

### 3.1 Light modulates cnidocyte discharge in distantly related cnidarians

**FIGURE 2:**
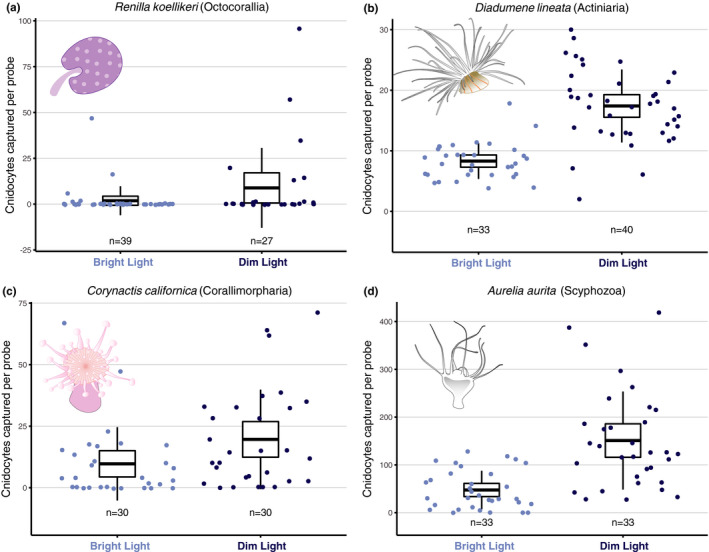
Cnidocyte discharge increases when polyps are exposed to dim blue light, a response conserved across long‐diverged cnidarian species. Under dim blue light (470 nm; 0.1 W/cm^2^), discharge of cnidocysts in the gelatin matrix was significantly higher than in bright blue light (470 nm; 2.8 W/cm^2^) assays (Wilcoxon Rank‐Sum Test, two‐tailed; *Aurelia*: *p* < 0.0001, *Corynactis*: *p* = 0.025, *Diadumene*: *p* < 0.0001, *Renilla*: *p* = 0.022; see *Materials and Methods* for details). Center lines in box plots correspond to the sample mean, top and bottom extremes represent upper and lower 95% confidence interval points, and whiskers are one standard deviation lines

**FIGURE 3:**
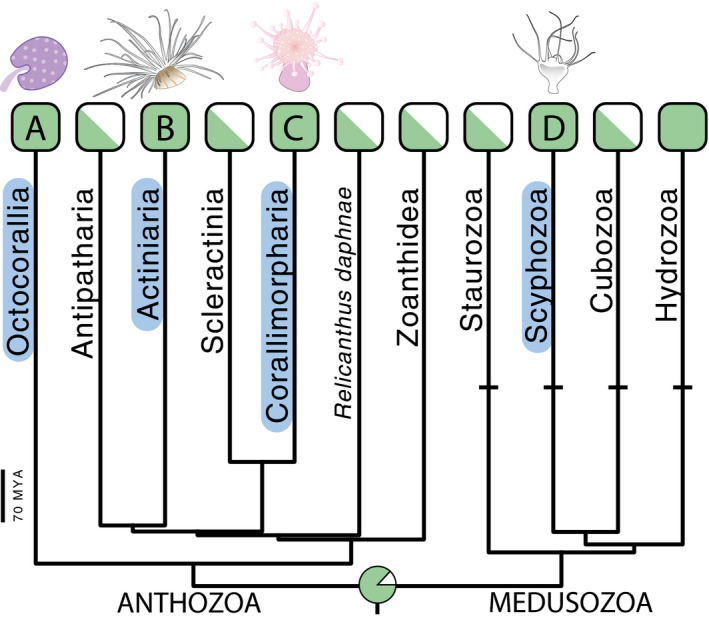
Maximum likelihood ancestral state reconstruction on the main phylogeny from Picciani et al. (2018). Marginal likelihoods of ancestral states (light modulated cnidocyte discharge present, green; absent, white) at the cnidarian ancestor node are shown in the pie chart and inferred with a symmetric Markov two‐state model (equal rates) of trait evolution. Letters and blue ovals show where studied species are placed in the phylogeny (A: *Renilla*, B: *Diadumene*, C: *Corynactis*, D: *Aurelia*). Tip states of groups for which we lack information on light modulated cnidocyte discharge are scored as missing data and shown as rectangles half colored in green. Horizontal bars indicate lineages in which eyes convergently evolved. Scale bar denotes time in millions of years. See Figure S1 for the whole phylogeny with ancestral states

Our analyses reveal a clear trend across distantly related cnidarians to use light for modulating the discharge of their cnidocytes (Figure 2) and indicate the cnidarian ancestor was also able to do so (Figure 3). Overall, the discharge of cnidocytes into probes was significantly higher for polyps exposed to a dim compared with bright blue LED light (Figure 2). Our statistical power was very high (~100%) for *Diadumene* and *Aurelia*, indicating that we can be very confident in the effect of light intensity on cnidocyte discharge in these two long‐diverged taxa (~700 mya). Conversely, power was lower for the other two taxa (*Renilla* and *Corynactis*; 40.3% and 52.9%, respectively) so that despite significant effects (*p* = 0.025 in *Corynactis*; *p* = 0.022 in *Renilla*), these should be considered with caution because low power may increase the chance of false positive results (Christley, 2010).

Our zero‐inflated negative binomial model agrees with our other statistical tests and additionally finds evidence for an overabundance of zeros in our count data, especially among dim conditions and within *Corynactis* and *Renilla* (Zero‐inflation model, *p* < 0.01 for each). This model was preferred to a simple negative binomial regression (Vuong test statistic for AIC values, *p* < 0.001), indicating that accounting for excess zeros in our cnidocyte firing is a better descriptor of our data than not. In this model, both species and experimental condition significantly explain variation in cnidocyte firing (Analysis of Deviance, *p* < 0.01 for each). Taken together, and as is most evident in distribution of our data (Figure 2), we believe that light modulation of cnidocyte firing is likely across these taxa.

### 3.2 Species‐specific variation in numbers of discharged cnidocytes

The octocoral *Renilla* discharged substantially fewer cnidocytes on average (from each treatment) than all other species, while the scyphopolyp *Aurelia* discharged more cnidocytes than the octocoral, the sea anemone *Diadumene* and the corallimorph *Corynactis*. That could be explained by either a comparable density of cnidocytes among species but differential use, variation of cnidocyte density in tentacles among species or a combination of both. For instance, octocorals often lack cnidocytes altogether, and other species of *Renilla* possess low numbers of a simple type of nematocyte (Mariscal & Bigger, 1977). In turn, scyphopolyps depend primarily on only one type of cnidocyte, the nematocyte, as opposed to most anthozoans, which use two types of cnidocytes (nematocytes and spirocytes) for lassoing prey (Fautin, 2009). That the scyphopolyp *Aurelia* relies only on nematocytes might explain its higher discharge averages compared with other species.

## 4 DISCUSSION

Our study presents empirical support for a sensory task that we suggest as a possible role for ancestral photoreceptors that predate cnidarian eyes. By testing whether the modulation of cnidocyte discharge by light occurs among long‐diverged cnidarian lineages and reconstructing the state of the cnidarian ancestor, we find support for the hypothesis that this light response is a deeply conserved sensory task preserved over millions of years. Because we find a broad diversity of cnidarian polyps discharge significantly more cnidocytes during exposure to dim blue light compared with bright blue light, we suggest that ancestral photoreceptors in Cnidaria regulated the discharge of cnidocytes. Several ecological reasons could explain why distantly related species tend to discharge more cnidocytes in dimmer light (see discussion in Plachetzki et al. 2012). For example, fine tuning cnidocyte discharge to maximize prey capture either at dusk, when zooplankton migrate to surface waters, or when prey items cast a shadow on the polyp. Although we do not rule out other possible light sensing functions, because cnidocyte discharge is still the primary means of defense and prey capture of almost all cnidarians, such a long‐standing photoreceptive function could have facilitated multiple convergent eye origins in the group by maintaining phototransduction pathways and enabling them to be later exploited for vision.

Organization of cnidocytes and their sensory apparatus vary extensively between cnidarian classes (Anderson & Bouchard, 2009), yet a similar innervation pattern (Anderson et al., 2004) suggests photoreceptor cells could still have persisted in the circuitry controlling cnidocyte discharge. Spatial positioning of cnidocytes in tentacles varies considerably—from patchy in hydrozoans and scyphozoans to uniform in sea anemones and corals (Anderson & Bouchard, 2009). Additionally, receptor complexes associated with cnidocytes can be produced solely by the cnidocytes themselves or receive projections from nearby ciliary cells (Watson & Mire‐Thibodeaux, 1994). Given such seemingly divergent organization, an alternative to homology of light modulation of cnidocyte discharge would be convergence of such light responsiveness via repeated co‐option of photoreceptor cells into cnidocyte circuitry. If convergent, the ancestral cnidocyte circuitry would have lacked photoreceptor cells, which would have been later independently assimilated into the circuitry of cnidocytes. But cnidarian photoreceptor cells are strongly peptidergic (Martin, 2002, 2004; Plickert & Schneider, 2004), and cnidocytes are innervated by networks of peptidergic neurons in all cnidarian classes regardless of their cnidocyte organization (Anderson et al., 2004; Westfall, 2004). These observations on peptidergic neurons, coupled with our inference that light modulation of cnidocyte discharge was ancestral, are consistent with a hypothesis that the cnidarian ancestor possessed photoreceptor cells that could send modulatory signals to cnidocytes and that these cells likely persisted in cnidocyte circuitry over evolutionary time.

Of the various light sensing genes in cnidarians, only xenopsins (called cnidops in cnidarians) are known to mediate photoreception in both Medusozoa and eyeless Anthozoa, suggesting that xenopsins could be used to sense light for cnidocyte discharge. For instance, different light sensing molecules, either nonopsin proteins or opsin types other than xenopsin, could be used for light detection in species of anthozoans. Even though anthozoans can sense light with cryptochromes and two opsin types besides xenopsin (Gornik *et al*., no date; Ramirez et al., 2016; Reitzel et al., 2013; Picciani et al., 2018), only the xenopsin seems to be used for light sensing by medusozoans. Interestingly, it is both the light sensitive molecule in photoreceptor cells of eyes and photosensory neurons that modulate the discharge of cnidocytes in *Hydra* (Plachetzki et al., 2012). It is likely that a homologous light response would be mediated by a light sensing molecule shared among all cnidarians, such as xenopsin. Demonstrating that the modulation of cnidocyte discharge in anthozoans is done with photoreceptors that use xenopsins would reinforce photoreceptor homology.

Other roles besides modulation of cnidocyte discharge are also possible for photoreceptors in the cnidarian ancestor, thought to be a solitary polyp lacking symbionts (Kayal et al., 2018). Several functions, including larval settlement and phototaxis, could also be ancestral—but we do not yet know whether they use opsins. If not opsin‐mediated, it seems unlikely such photoreceptors became assimilated into eyes that invariably use opsin. A topic for future research would be to test whether other light‐dependent functions are mediated by opsins, and if so, whether the functions are ancestral in Cnidaria. Second, opsin‐expressing ectodermal cells in the gonads of *Clytia* control oocyte maturation (Quiroga Artigas et al., 2018), so that spawning is another candidate for an ancestral photoreceptive function in cnidarians. Testing whether light‐influenced spawning is ancestral would require a survey of other species besides *Clytia*. A broad survey could be facilitated by the many available reports of light‐influenced spawning in Cnidaria (see item S1 in Picciani et al., 2018). Understanding the phototransduction pathways underlying spawning across species using genetic and experimental approaches would also be important to uncover the identity of photoreceptor cells and their relationship to eye precursors.

In addition to photoreceptor cells, other key precursor modules like pigments and crystallins probably predated cnidarian eye origins and served other organismal functions prior to visual function. For instance, one module—the biosynthesis machinery of melanin that includes tyrosinases—is present in species of both Anthozoa and Medusozoa (Dunlap et al., 2013; Esposito et al., 2012), the two major cnidarian sister lineages, and therefore could also be ancestral. Melanin synthesis is involved in many biological processes outside of cnidarian eyes, including functioning as a trigger for scyphopolyps to strobilate and produce jellyfish (Berking et al., 2005; Van den Branden et al., 1980; Van den Branden, Van den Sande, & Decleir, 1980). Moreover, melanin is also used by corals, sea fans, and anemones to create a physical barrier against pathogens, and melanin synthesis is correlated with disease resistance in corals (Mydlarz et al., 2008; Mydlarz & Palmer, 2011; Palmer et al., 2008, 2012; Petes et al., 2003; Zaragoza et al., 2014). Another precursor module, the crystallin proteins, forms lenses in the eyes of box jellyfish and may be derived from proteins with nonoptical functions (Piatigorsky et al., 1989, 2001; Piatigorsky et al., 1993). We know relatively little about the origins, both structural and functional, of box jellyfish lens crystallins, though they are thought to be closely related to vertebrate saposins (Piatigorsky et al., 2001). Crystallin homologs seem to occur in sea anemones (Nicosia et al., 2014) and could perhaps be present in other lineages of eyeless cnidarians or could have occurred ancestrally and been lost in most eyeless species.

By testing a wide breadth of cnidarian diversity for a light‐influenced response known to involve a family of opsins used for vision, our results highlight one possible early role for eye precursors in Cnidaria was to modulate cnidocyte discharge. These results contribute to our understanding of eye evolution by using a phylogenetic context to propose an explanation for where the photoreceptor cells of eyes come from, and what functions they possibly had before becoming functionally integrated with other structures to mediate vision. It also raises interesting questions about how sensory tasks continued to evolve in lineages that acquired eyes. Which novel functions were cnidarians able to perform once they evolved directional photoreceptors and image‐forming eyes? Did those new functions supersede ancestral functions? As proposed by Nilsson (2013), the evolution of increasingly complex visual tasks can be studied concomitantly with eye morphology so we can understand evolutionary trajectories accompanying both function and structure. By advancing a possible ancient role for cnidarian eye precursors, our study helps to start dissecting the functional drivers that can elaborate morphological complexity.

## CONFLICT OF INTEREST

The authors declare no conflicts of interest.

## AUTHOR CONTRIBUTIONS

**Natasha Picciani:** Conceptualization (equal); Data curation (lead); Formal analysis (equal); Funding acquisition (equal); Investigation (equal); Methodology (equal); Visualization (equal); Writing‐original draft (lead); Writing‐review & editing (equal). **Jamie R. Kerlin:** Investigation (equal); Writing‐review & editing (equal). **Katia Jindrich:** Investigation (equal); Writing‐review & editing (equal). **Nicholai M. Hensley:** Formal analysis (equal); Visualization (equal); Writing‐review & editing (equal). **David A. Gold:** Investigation (equal); Writing‐review & editing (equal). **Todd H. Oakley:** Conceptualization (equal); Funding acquisition (equal); Methodology (equal); Resources (lead); Writing‐original draft (supporting); Writing‐review & editing (equal).

## ETHICAL APPROVAL

The authors followed all guidelines for ethical treatment of the animals.

### OPEN RESEARCH BADGES

This article has earned an Open Data Badge for making publicly available the digitally‐shareable data necessary to reproduce the reported results. The data is available at https://doi.org/10.5061/dryad.9w0vt4bds.

## Supporting information

Figure S1

## Data Availability

Raw datasets and analysis code are deposited in the Dryad repository https://doi.org/10.5061/dryad.9w0vt4bds.
