# Supplementary material for: Light modulated cnidocyte discharge predates the origins of eyes in Cnidaria
Source: Ecol Evol. 2021 Mar 17;11(9):3933–40. doi: 10.1002/ece3.7280 (PMC8093662; doi:10.1002/ece3.7280)
Supplement: Supplementary file 1 — Figure S1 [file ECE3-11-3933-s001.pdf]

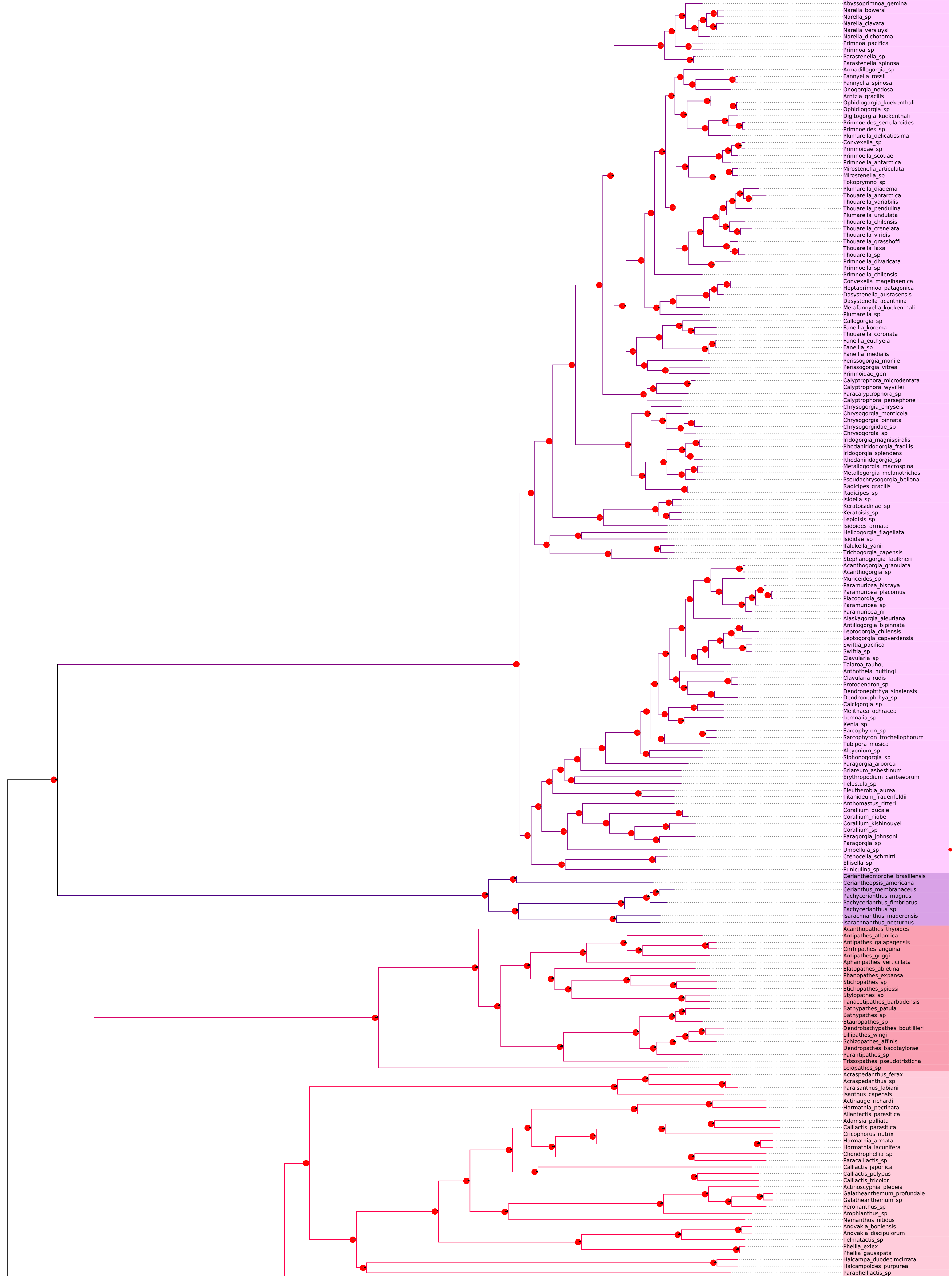

Abyssoprimnoa_gemina
Narella_bowersi
Narella_sp
Narella_clavata
Narella_versluysi
Narella_dichotoma
Primnoa_pacifica
Primnoa_sp
Parastenella_sp
Parastenella_spinosa
Armadillogorgia_sp
Fannyella_rossii
Fannyella_spinosa
Onogorgia_nodosa
Arntzia_gracilis
Ophidiogorgia_kuekenthali
Ophidiogorgia_sp
Digitogorgia_kuekenthali
Primnoeides_sertularoides
Primnoeides_sp
Plumarella_delicatissima
Convexella_sp
Primnoidae_sp
Primnoella_scotiae
Primnoella_antarctica
Mirostenella_articulata
Mirostenella_sp
Tokoprymno_sp
Plumarella_diadema
Thouarella_antarctica
Thouarella_variabilis
Thouarella_pendulina
Plumarella_undulata
Thouarella_chilensis
Thouarella_crenelata
Thouarella_viridis
Thouarella_grasshoffi
Thouarella_laxa
Thouarella_sp
Primnoella_divaricata
Primnoella_sp
Primnoella_chilensis
Convexella_magelhaenica
Heptaprimnoa_patagonica
Dasystenella_austasensis
Dasystenella_acanthina
Metafannyella_kuekenthali
Plumarella_sp
Callogorgia_sp
Fanellia_korema
Thouarella_coronata
Fanellia_euthyeia
Fanellia_sp
Fanellia_medialis
Perissogorgia_monile
Perissogorgia_vitrea
Primnoidae_gen
Calyptrophora_microdentata
Calyptrophora_wyvillei
Paracalyptrophora_sp
Calyptrophora_persephone
Chrysogorgia_chryseis
Chrysogorgia_monticola
Chrysogorgia_pinnata
Chrysogorgiidae_sp
Chrysogorgia_sp
Iridogorgia_magnispiralis
Rhodaniridogorgia_fragilis
Iridogorgia_splendens
Rhodaniridogorgia_sp
Metallogorgia_macrospina
Metallogorgia_melanotrichos
Pseudochrysogorgia_bellona
Radicipes_gracilis
Radicipes_sp
Isidella_sp
Keratoisidinae_sp
Keratoisis_sp
Lepidisis_sp
Isidoides_armata
Helicogorgia_flagellata
Isididae_sp
Ifalukella_yanii
Trichogorgia_capensis
Stephanogorgia_faulkneri
Acanthogorgia_granulata
Acanthogorgia_sp
Muriceides_sp
Paramuricea_biscaya
Paramuricea_placomus
Placogorgia_sp
Paramuricea_sp
Paramuricea_nr
Alaskagorgia_aleutiana
Antillogorgia_bipinnata
Leptogorgia_chilensis
Leptogorgia_capverdensis
Swiftia_pacifica
Swiftia_sp
Clavularia_sp
Taiaroa_tauhou
Anthothela_nuttingi
Clavularia_rudis
Protodendron_sp
Dendronephthya_sinaiensis
Dendronephthya_sp
Calcigorgia_sp
Melithaea_ochracea
Lemnalia_sp
Xenia_sp
Sarcophyton_sp
Sarcophyton_trocheliophorum
Tubipora_musica
Alcyonium_sp
Siphonogorgia_sp
Paragorgia_arborea
Briareum_asbestinum
Erythropodium_caribaeorum
Telestula_sp
Eleutherobia_aurea
Titanideum_frauenfeldii
Anthomastus_ritteri
Corallium_ducale
Corallium_niobe
Corallium_kishinouyei
Corallium_sp
Paragorgia_johnsoni
Paragorgia_sp
Umbellula_sp
Ctenocella_schmitti
Ellisella_sp
Funiculina_sp
Ceriantheomorphe_brasiliensis
Ceriantheopsis_americana
Cerianthus_membranaceus
Pachycerianthus_magnus
Pachycerianthus_fimbriatus
Pachycerianthus_sp
Isarachnanthus_maderensis
Isarachnanthus_nocturnus
Acanthopathes_thyoides
Antipathes_atlantica
Antipathes_galapagensis
Cirrhipathes_anguina
Antipathes_griggi
Aphanipathes_verticillata
Elatopathes_abietina
Phanopathes_expansa
Stichopathes_sp
Stichopathes_spiessi
Stylopathes_sp
Tanacetipathes_barbadensis
Bathypathes_patula
Bathypathes_sp
Stauropathes_sp
Dendrobathypathes_boutillieri
Lillipathes_wingi
Schizopathes_affinis
Dendropathes_bacotaylorae
Parantipathes_sp
Trissopathes_pseudotristicha
Leiopathes_sp
Acraspedanthus_ferax
Acraspedanthus_sp
Paraisanthus_fabiani
Isanthus_capensis
Actinauge_richardi
Hormathia_pectinata
Allantactis_parasitica
Adamsia_palliata
Calliactis_parasitica
Cricophorus_nutrix
Hormathia_armata
Hormathia_lacunifera
Chondrophellia_sp
Paracalliactis_sp
Calliactis_japonica
Calliactis_polypus
Calliactis_tricolor
Actinoscyphia_plebeia
Galatheanthemum_profundale
Galatheanthemum_sp
Peronanthus_sp
Amphianthus_sp
Nemanthus_nitidus
Andvakia_boniensis
Andvakia_discipulorum
Telmatactis_sp
Phellia_exlex
Phellia_gausapata
Halcampa_duodecimcirrata
Halcampoides_purpurea
Paraphelliactis_sp

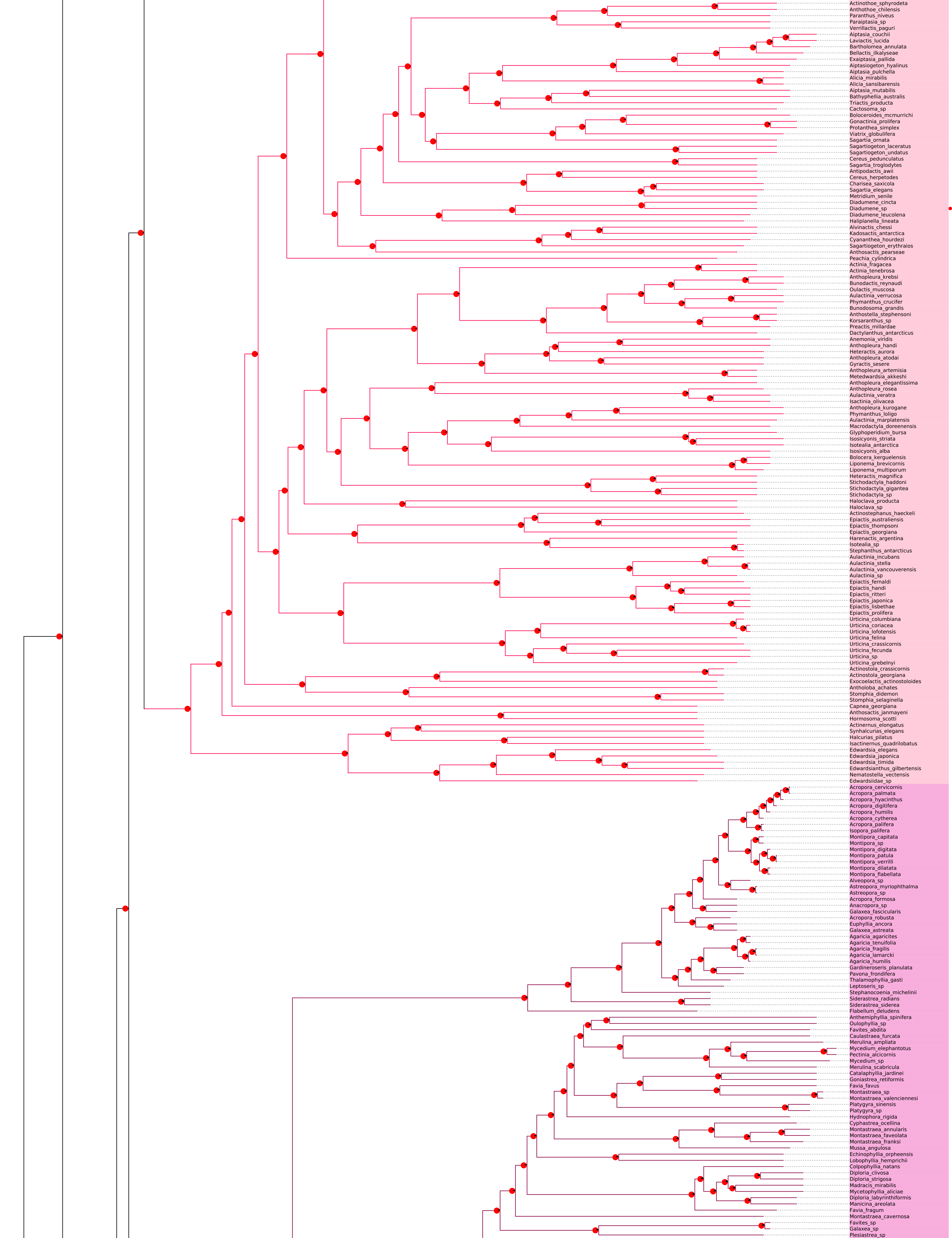

Actinothoe_sphyrodeta
Anthothoe_chilensis
Paranthus_niveus
Paraiptasia_sp
Verrillactis_paguri
Aiptasia_couchii
Laviactis_lucida
Bartholomea_annulata
Bellactis_ilkalyseae
Exaiptasia_pallida
Aiptasiogeton_hyalinus
Aiptasia_pulchella
Alicia_mirabilis
Alicia_sansibarensis
Aiptasia_mutabilis
Bathyphellia_australis
Triactis_producta
Cactosoma_sp
Boloceroides_mcmurrichi
Gonactinia_prolifera
Protanthea_simplex
Viatrix_globulifera
Sagartia_ornata
Sagartiogeton_laceratus
Sagartiogeton_undatus
Cereus_pedunculatus
Sagartia_troglodytes
Antipodactis_awii
Cereus_herpetodes
Charisea_saxicola
Sagartia_elegans
Metridium_senile
Diadumene_cincta
Diadumene_sp
Diadumene_leucolena
Haliplanella_lineata
Alvinactis_chessi
Kadosactis_antarctica
Cyananthea_hourdezi
Sagartiogeton_erythraios
Anthosactis_pearseae
Peachia_cylindrica
Actinia_fragacea
Actinia_tenebrosa
Anthopleura_krebsi
Bunodactis_reynaudi
Oulactis_muscosa
Aulactinia_verrucosa
Phymanthus_crucifer
Bunodosoma_grandis
Anthostella_stephensoni
Korsaranthus_sp
Preactis_millardae
Dactylanthus_antarcticus
Anemonia_viridis
Anthopleura_handi
Heteractis_aurora
Anthopleura_atodai
Gyractis_sesere
Anthopleura_artemisia
Metedwardsia_akkeshi
Anthopleura_elegantissima
Anthopleura_rosea
Aulactinia_veratra
Isactinia_olivacea
Anthopleura_kurogane
Phymanthus_loligo
Aulactinia_marplatensis
Macrodactyla_doreenensis
Glyphoperidium_bursa
Isosicyonis_striata
Isotealia_antarctica
Isosicyonis_alba
Bolocera_kerguelensis
Liponema_brevicornis
Liponema_multiporum
Heteractis_magnifica
Stichodactyla_haddoni
Stichodactyla_gigantea
Stichodactyla_sp
Haloclava_producta
Haloclava_sp
Actinostephanus_haeckeli
Epiactis_australiensis
Epiactis_thompsoni
Epiactis_georgiana
Harenactis_argentina
Isotealia_sp
Stephanthus_antarcticus
Aulactinia_incubans
Aulactinia_stella
Aulactinia_vancouverensis
Aulactinia_sp
Epiactis_fernaldi
Epiactis_handi
Epiactis_ritteri
Epiactis_japonica
Epiactis_lisbethae
Epiactis_prolifera
Urticina_columbiana
Urticina_coriacea
Urticina_lofotensis
Urticina_felina
Urticina_crassicornis
Urticina_fecunda
Urticina_sp
Urticina_grebelnyi
Actinostola_crassicornis
Actinostola_georgiana
Exocoelactis_actinostoloides
Antholoba_achates
Stomphia_didemon
Stomphia_selaginella
Capnea_georgiana
Anthosactis_janmayeni
Hormosoma_scotti
Actinernus_elongatus
Synhalcurias_elegans
Halcurias_pilatus
Isactinernus_quadrilobatus
Edwardsia_elegans
Edwardsia_japonica
Edwardsia_timida
Edwardsianthus_gilbertensis
Nematostella_vectensis
Edwardsiidae_sp
Acropora_cervicornis
Acropora_palmata
Acropora_hyacinthus
Acropora_digitifera
Acropora_humilis
Acropora_cytherea
Acropora_palifera
Isopora_palifera
Montipora_capitata
Montipora_sp
Montipora_digitata
Montipora_patula
Montipora_verrilli
Montipora_dilatata
Montipora_flabellata
Alveopora_sp
Astreopora_myriophthalma
Astreopora_sp
Acropora_formosa
Anacropora_sp
Galaxea_fascicularis
Acropora_robusta
Euphyllia_ancora
Galaxea_astreata
Agaricia_agaricites
Agaricia_tenuifolia
Agaricia_fragilis
Agaricia_lamarcki
Agaricia_humilis
Gardineroseris_planulata
Pavona_frondifera
Thalamophyllia_gasti
Leptoseris_sp
Stephanocoenia_michelinii
Siderastrea_radians
Siderastrea_siderea
Flabellum_deludens
Anthemiphyllia_spinifera
Oulophyllia_sp
Favites_abdita
Caulastraea_furcata
Merulina_ampliata
Mycedium_elephantotus
Pectinia_alcicornis
Mycedium_sp
Merulina_scabricula
Cataphyllia_jardinei
Goniastrea_retiformis
Favia_favus
Montastraea_sp
Montastraea_valenciennesi
Platygyra_sinensis
Platygyra_sp
Hydnophora_rigida
Cyphastrea_ocellina
Montastraea_annularis
Montastraea_faveolata
Montastraea_franksi
Mussa_angulosa
Echinophyllia_orpheensis
Lobophyllia_hemprichii
Colpophyllia_natans
Diploria_clivosa
Diploria_strigosa
Madracis_mirabilis
Mycetophyllia_aliciae
Diploria_labyrinthiformis
Manicina_areolata
Favia_fragum
Montastraea_cavernosa
Favites_sp
Galaxea_sp
Plesiastrea_sp

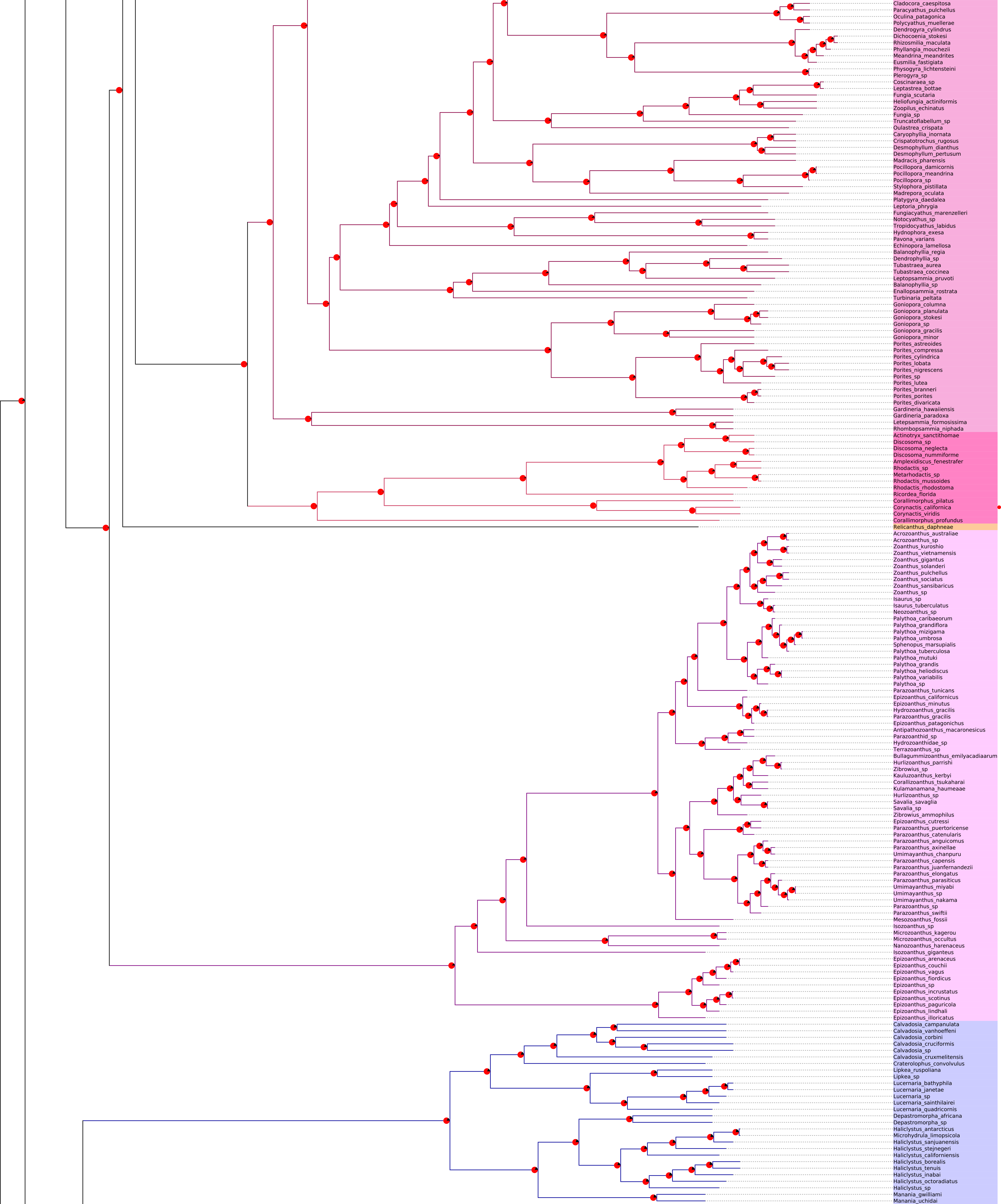
Cladocora_caespitosa
Paracyathus_pulchellus
Oculina_patagonica
Polycyathus_muellerae
Dendrogyra_cylindrus
Dichocoenia_stokesi
Rhizosmilia_maculata
Phyllangia_mouchezii
Meandrina_meandrites
Eusmilia_fastigiata
Physogyra_lichtensteini
Plerogyra_sp
Coscinaraea_sp
Leptastrea_bottae
Fungia_scutaria
Heliofungia_actiniformis
Zoopilus_echinatus
Fungia_sp
Truncatoflabellum_sp
Oulastrea_crispata
Caryophyllia_inornata
Crispatotrochus_rugosus
Desmophyllum_dianthus
Desmophyllum_pertusum
Madracis_pharensis
Pocillopora_damicornis
Pocillopora_meandrina
Pocillopora_sp
Stylophora_pistillata
Madrepora_oculata
Platygyra_daedalea
Leptoria_phrygia
Fungiacyathus_marenzelleri
Notocyathus_sp
Tropidocyathus_labidus
Hydnophora_exesa
Pavona_varians
Echinopora_lamellosa
Balanophyllia_regia
Dendrophyllia_sp
Tubastraea_aurea
Tubastraea_coccinea
Leptopsammia_pruvoti
Balanophyllia_sp
Enallopsammia_rostrata
Turbinaria_peltata
Goniopora_columna
Goniopora_planulata
Goniopora_stokesi
Goniopora_sp
Goniopora_gracilis
Goniopora_minor
Porites_astreoides
Porites_compressa
Porites_cylindrica
Porites_lobata
Porites_nigrescens
Porites_sp
Porites_lutea
Porites_branneri
Porites_porites
Porites_divaricata
Gardineria_hawaiiensis
Gardineria_paradoxa
Letepsammia_formosissima
Rhombopsammia_niphada
Actinotryx_sanctithomae
Discosoma_sp
Discosoma_neglecta
Discosoma_nummiforme
Amplexidiscus_fenestrafer
Rhodactis_sp
Metarhodactis_sp
Rhodactis_mussoides
Rhodactis_rhodostoma
Ricordea_florida
Corallimorphus_pilatus
Corynactis_californica
Corynactis_viridis
Corallimorphus_profundus
Relicanthus_daphneae
Acrozoanthus_australiae
Acrozoanthus_sp
Zoanthus_kuroshio
Zoanthus_vietnamensis
Zoanthus_gigantus
Zoanthus_solanderi
Zoanthus_pulchellus
Zoanthus_sociatus
Zoanthus_sansibaricus
Zoanthus_sp
Isaurus_sp
Isaurus_tuberculatus
Neozoanthus_sp
Palythoa_caribaeorum
Palythoa_grandiflora
Palythoa_mizigama
Palythoa_umbrosa
Sphenopus_marsupialis
Palythoa_tuberculosa
Palythoa_mutuki
Palythoa_grandis
Palythoa_heliodiscus
Palythoa_variabilis
Palythoa_sp
Parazoanthus_tunicans
Epizoanthus_californicus
Epizoanthus_minutus
Hydrozoanthus_gracilis
Parazoanthus_gracilis
Epizoanthus_patagonichus
Antipathozoanthus_macaronesicus
Parazoanthid_sp
Hydrozoanthidae_sp
Terrazoanthus_sp
Bullagummizoanthus_emilyacadiaarum
Hurlizoanthus_parrishi
Zibrowius_sp
Kauluzoanthus_kerbyi
Corallizoanthus_tsukaharai
Kulamanamana_haumeaae
Hurlizoanthus_sp
Savalia_savaglia
Savalia_sp
Zibrowius_ammophilus
Epizoanthus_cutressi
Parazoanthus_puertoricense
Parazoanthus_catenularis
Parazoanthus_anguicomus
Parazoanthus_axinellae
Umimayanthus_chanpuru
Parazoanthus_capensis
Parazoanthus_juanfernandezii
Parazoanthus_elongatus
Parazoanthus_parasiticus
Umimayanthus_miyabi
Umimayanthus_sp
Umimayanthus_nakama
Parazoanthus_sp
Parazoanthus_swiftii
Mesozoanthus_fossii
Isozoanthus_sp
Microzoanthus_kagerou
Microzoanthus_occultus
Nanozoanthus_harenaceus
Isozoanthus_giganteus
Epizoanthus_arenaceus
Epizoanthus_couchii
Epizoanthus_vagus
Epizoanthus_fiordicus
Epizoanthus_sp
Epizoanthus_incrustatus
Epizoanthus_scotinus
Epizoanthus_paguricola
Epizoanthus_lindhali
Epizoanthus_illoricatus
Calvadosia_campanulata
Calvadosia_vanhoeffeni
Calvadosia_corbini
Calvadosia_cruciformis
Calvadosia_sp
Calvadosia_cruxmelitensis
Craterolophus_convolvulus
Lipkea_ruspoliana
Lipkea_sp
Lucernaria_bathyphila
Lucernaria_janetae
Lucernaria_sp
Lucernaria_sainthilairei
Lucernaria_quadricornis
Depastromorpha_africana
Depastromorpha_sp
Haliclystus_antarcticus
Microhydrula_limopsicola
Haliclystus_sanjuanensis
Haliclystus_stejnegeri
Haliclystus_californiensis
Haliclystus_borealis
Haliclystus_tenuis
Haliclystus_inabai
Haliclystus_octoradiatus
Haliclystus_sp
Manania_gwilliami
Manania_uchidai

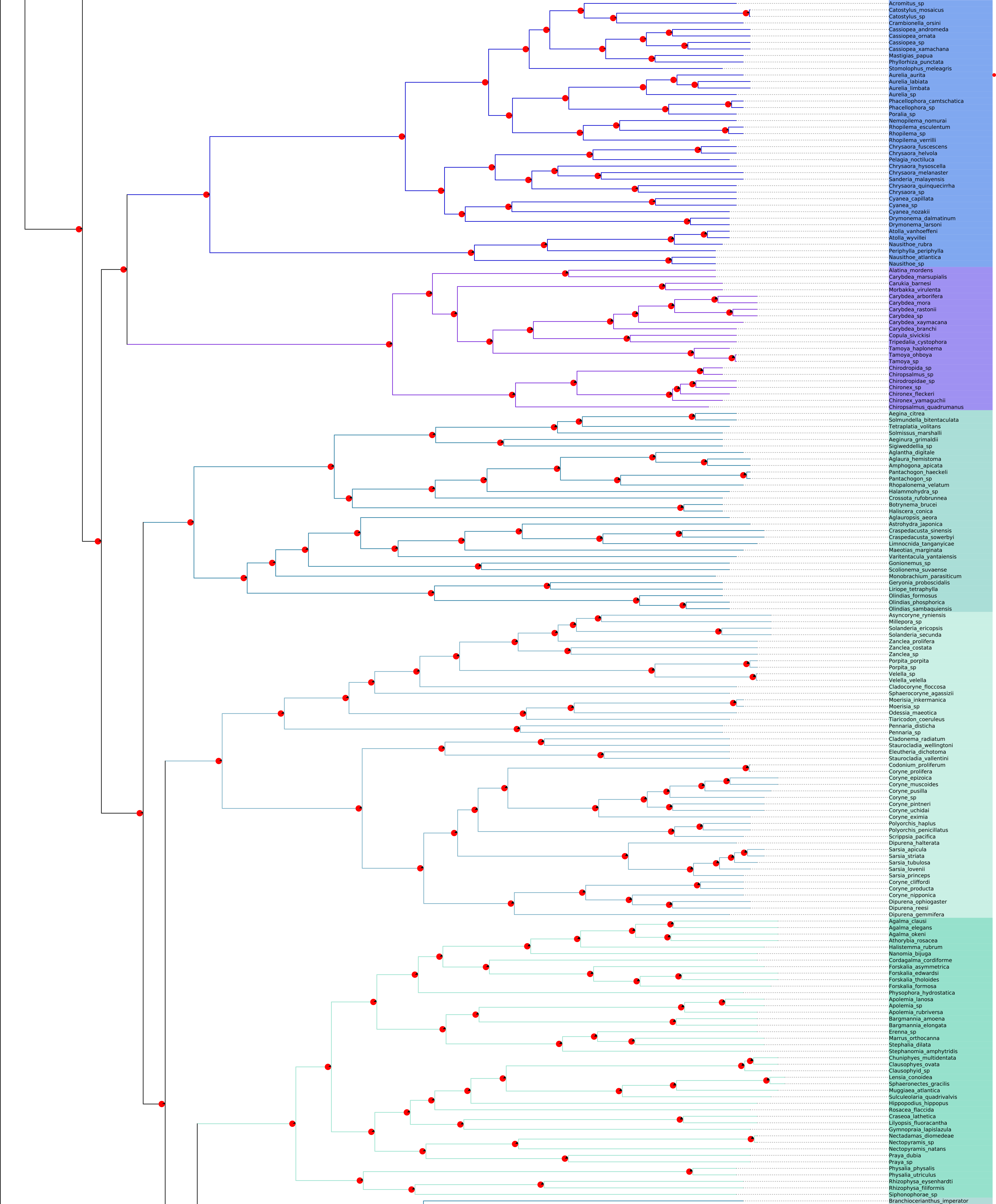

Acromitus_sp
Catostylus_mosaicus
Catostylus_sp
Crambionella_orsini
Cassiopea_andromeda
Cassiopea_ornata
Cassiopea_sp
Cassiopea_xamachana
Mastigias_papua
Phyllorhiza_punctata
Stomolophus_meleagris
Aurelia_aurita
Aurelia_labiata
Aurelia_limbata
Aurelia_sp
Phacellophora_camtschatica
Phacellophora_sp
Poralia_sp
Nemopilema_nomurai
Rhopilema_esculentum
Rhopilema_sp
Rhopilema_verrilli
Chrysaora_fuscescens
Chrysaora_helvola
Pelagia_noctiluca
Chrysaora_hysoscella
Chrysaora_melanaster
Sanderia_malayensis
Chrysaora_quinquecirrha
Chrysaora_sp
Cyanea_capillata
Cyanea_sp
Cyanea_nozakii
Drymonema_dalmatinum
Drymonema_larsoni
Atolla_vanhoeffeni
Atolla_wyvillei
Nausithoe_rubra
Periphylla_periphylla
Nausithoe_atlantica
Nausithoe_sp
Alatina_mordens
Carybdea_marsupialis
Carukia_barnesi
Morbakka_virulenta
Carybdea_arborifera
Carybdea_mora
Carybdea_rastonii
Carybdea_sp
Carybdea_xaymacana
Carybdea_branchi
Copula_sivickisi
Tripedalia_cystophora
Tamoya_haplonema
Tamoya_ohboya
Tamoya_sp
Chirodropida_sp
Chiropsalmus_sp
Chirodropidae_sp
Chironex_sp
Chironex_fleckeri
Chironex_yamaguchii
Chiropsalmus_quadrumanus
Aegina_citrea
Solmundella_bitentaculata
Tetraplatia_volitans
Solmissus_marshalli
Aeginura_grimaldii
Sigiweddellia_sp
Aglantha_digitale
Aglaura_hemistoma
Amphogona_apicata
Pantachogon_haeckeli
Pantachogon_sp
Rhopalonema_velatum
Halammohydra_sp
Crossota_rufobrunnea
Botrynema_brucei
Haliscera_conica
Aglauropsis_aeora
Astrohydra_japonica
Craspedacusta_sinensis
Craspedacusta_sowerbyi
Limnocnida_tanganyicae
Maeotias_marginata
Varitentacula_yantaiensis
Gonionemus_sp
Scolionema_suvaense
Monobrachium_parasiticum
Geryonia_proboscidalis
Liriope_tetraphylla
Olindias_formosus
Olindias_phosphorica
Olindias_sambaquiensis
Asyncoryne_ryniensis
Millepora_sp
Solanderia_ericopsis
Solanderia_secunda
Zanclea_prolifera
Zanclea_costata
Zanclea_sp
Porpita_porpita
Porpita_sp
Velella_sp
Velella_velella
Cladocoryne_floccosa
Sphaerocoryne_agassizii
Moerisia_inkermanica
Moerisia_sp
Odessia_maeotica
Tiaricodon_coeruleus
Pennaria_disticha
Pennaria_sp
Cladonema_radiatum
Staurocladia_wellingtoni
Eleutheria_dichotoma
Staurocladia_vallentini
Codonium_proliferum
Coryne_prolifera
Coryne_epizoica
Coryne_muscoides
Coryne_pusilla
Coryne_sp
Coryne_pintneri
Coryne_uchidai
Coryne_eximia
Polyorchis_haplus
Polyorchis_penicillatus
Scrippsia_pacifica
Dipurena_halterata
Sarsia_apicula
Sarsia_striata
Sarsia_tubulosa
Sarsia_lovenii
Sarsia_princeps
Coryne_cliffordi
Coryne_producta
Coryne_nipponica
Dipurena_ophiogaster
Dipurena_reesi
Dipurena_gemmifera
Agalma_clausi
Agalma_elegans
Agalma_okeni
Athorybia_rosacea
Halistemma_rubrum
Nanomia_bijuga
Cordagalma_cordiforme
Forskalia_asymmetrica
Forskalia_edwardsi
Forskalia_tholoides
Forskalia_formosa
Physophora_hydrostatica
Apolemia_lanosa
Apolemia_sp
Apolemia_rubriversa
Bargmannia_amoena
Bargmannia_elongata
Erenna_sp
Marrus_orthocanna
Stephalia_dilata
Stephanomia_amphytridis
Chuniphyes_multidentata
Clausophyes_ovata
Clausophyid_sp
Lensia_conoidea
Sphaeronectes_gracilis
Muggiaea_atlantica
Sulculeolaria_quadrivalvis
Hippopodius_hippopus
Rosacea_flaccida
Craseoa_lathetica
Lilyopsis_fluoracantha
Gymnopraia_lapislazula
Nectadamas_diomedeae
Nectopyramis_sp
Nectopyramis_natans
Praya_dubia
Praya_sp
Physalia_physalis
Physalia_utriculus
Rhizophysa_eysenhardti
Rhizophysa_filiformis
Siphonophorae_sp
Branchiocerianthus_imperator

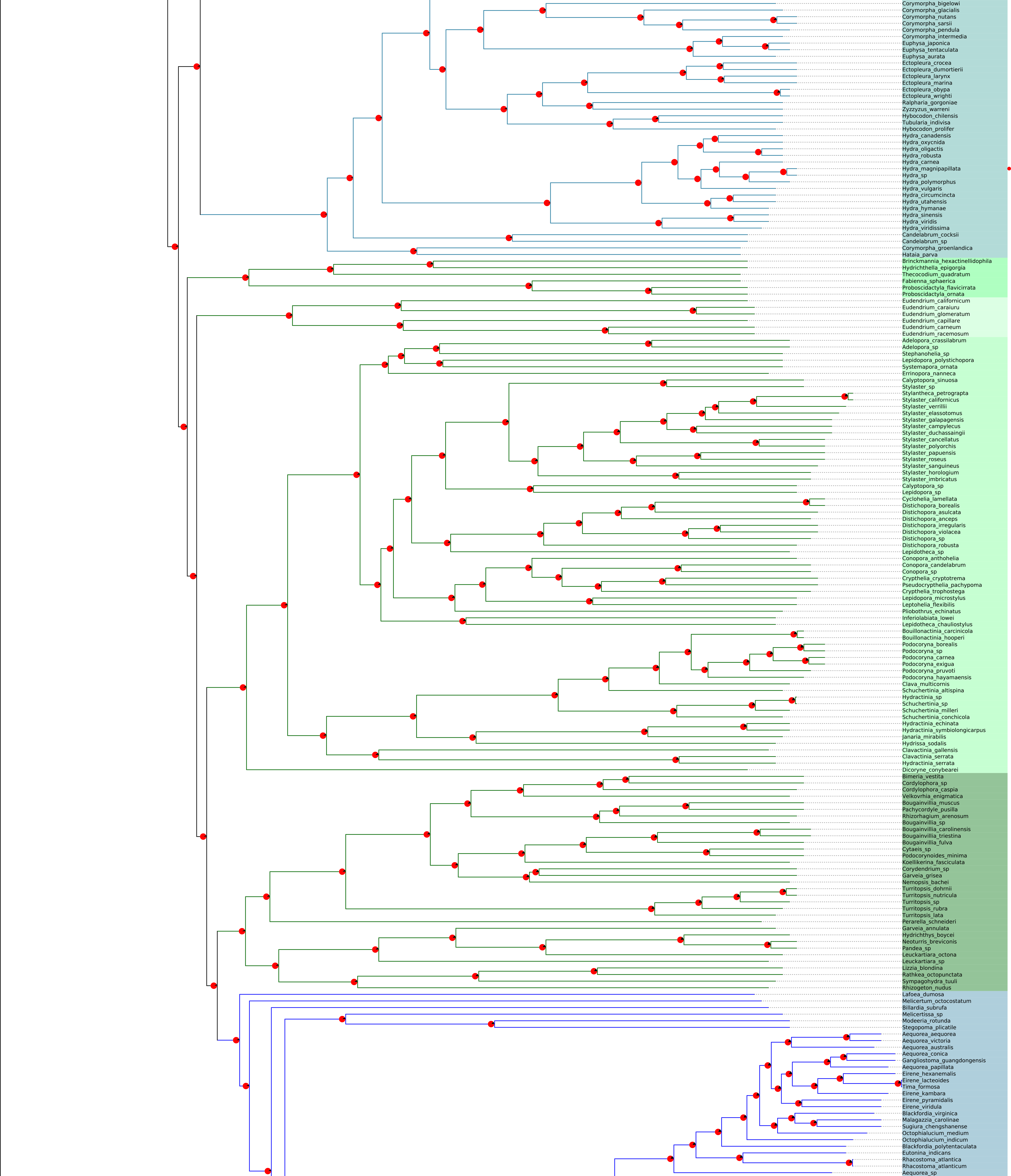
Corymorpha_bigelowi
Corymorpha_glacialis
Corymorpha_nutans
Corymorpha_sarsii
Corymorpha_pendula
Corymorpha_intermedia
Euphysa_japonica
Euphysa_tentaculata
Euphysa_aurata
Ectopleura_crocea
Ectopleura_dumortierii
Ectopleura_larynx
Ectopleura_marina
Ectopleura_obypa
Ectopleura_wrighti
Ralpharia_gorgoniae
Zyzzyzus_warreni
Hybocodon_chilensis
Tubularia_indivisa
Hybocodon_prolifer
Hydra_canadensis
Hydra_oxycnida
Hydra_oligactis
Hydra_robusta
Hydra_carnea
Hydra_magnipapillata
Hydra_sp
Hydra_polymorphus
Hydra_vulgaris
Hydra_circumcincta
Hydra_utahensis
Hydra_hymanae
Hydra_sinensis
Hydra_viridis
Hydra_viridissima
Candelabrum_cocksii
Candelabrum_sp
Corymorpha_groenlandica
Hataia_parva
Brinckmannia_hexactinellidophila
Hydrichthella_epigorgia
Thecocodium_quadratum
Fabienna_sphaerica
Proboscidactyla_flavicirrata
Proboscidactyla_ornata
Eudendrium_californicum
Eudendrium_caraiuru
Eudendrium_glomeratum
Eudendrium_capillare
Eudendrium_carneum
Eudendrium_racemosum
Adelopora_crassilabrum
Adelopora_sp
Stephanohelia_sp
Lepidopora_polystichopora
Systemapora_ornata
Errinopora_nanneca
Calyptopora_sinuosa
Stylaster_sp
Stylantheca_petrograpta
Stylaster_californicus
Stylaster_verrillii
Stylaster_elassotomus
Stylaster_galapagensis
Stylaster_campylecus
Stylaster_duchassaingii
Stylaster_cancellatus
Stylaster_polyorchis
Stylaster_papuensis
Stylaster_roseus
Stylaster_sanguineus
Stylaster_horologium
Stylaster_imbricatus
Calyptopora_sp
Lepidopora_sp
Cyclohelia_lamellata
Distichopora_borealis
Distichopora_asulcata
Distichopora_anceps
Distichopora_irregularis
Distichopora_violacea
Distichopora_sp
Distichopora_robusta
Lepidotheca_sp
Conopora_anthohelia
Conopora_candelabrum
Conopora_sp
Crypthelia_cryptotrema
Pseudocrypthelia_pachypoma
Crypthelia_trophostega
Lepidopora_microstylus
Leptohelia_flexibilis
Pliobothrus_echinatus
Inferiolabiata_lowei
Lepidotheca_chauliostylus
Bouillonactinia_carcinicola
Bouillonactinia_hooperi
Podocoryna_borealis
Podocoryna_sp
Podocoryna_carnea
Podocoryna_exigua
Podocoryna_pruvoti
Podocoryna_hayamaensis
Clava_multicornis
Schuchertinia_altispina
Hydractinia_sp
Schuchertinia_sp
Schuchertinia_milleri
Schuchertinia_conchicola
Hydractinia_echinata
Hydractinia_symbiolongicarpus
Janaria_mirabilis
Hydrissa_sodalis
Clavactinia_gallensis
Clavactinia_serrata
Hydractinia_serrata
Dicoryne_conybearei
Bimeria_vestita
Cordylophora_sp
Cordylophora_caspia
Velkovrhia_enigmatica
Bougainvillia_muscus
Pachycordyle_pusilla
Rhizorhagium_arenosum
Bougainvillia_sp
Bougainvillia_carolinensis
Bougainvillia_triestina
Bougainvillia_fulva
Cytaeis_sp
Podocorynoides_minima
Koellikerina_fasciculata
Corydendrium_sp
Garveia_grisea
Nemopsis_bachei
Turritopsis_dohrnii
Turritopsis_nutricula
Turritopsis_sp
Turritopsis_rubra
Turritopsis_lata
Perarella_schneideri
Garveia_annulata
Hydrichthys_boycei
Neoturris_breviconis
Pandea_sp
Leuckartiara_octona
Leuckartiara_sp
Lizzia_blondina
Rathkea_octopunctata
Sympagohydra_tuuli
Rhizogeton_nudus
Lafoea_dumosa
Melicertum_octocostatum
Billardia_subrufa
Melicertissa_sp
Modeeria_rotunda
Stegopoma_plicatile
Aequorea_aequorea
Aequorea_victoria
Aequorea_australis
Aequorea_conica
Gangliostoma_guangdongensis
Aequorea_papillata
Eirene_hexanemalis
Eirene_lacteoides
Tima_formosa
Eirene_kambara
Eirene_pyramidalis
Eirene_viridula
Blackfordia_virginica
Malagazzia_carolinae
Sugiura_chengshanensis
Octophialucium_medium
Octophialucium_indicum
Blackfordia_polytentaculata
Eutonina_indicans
Rhacostoma_atlantica
Rhacostoma_atlanticum
Aequorea_sp

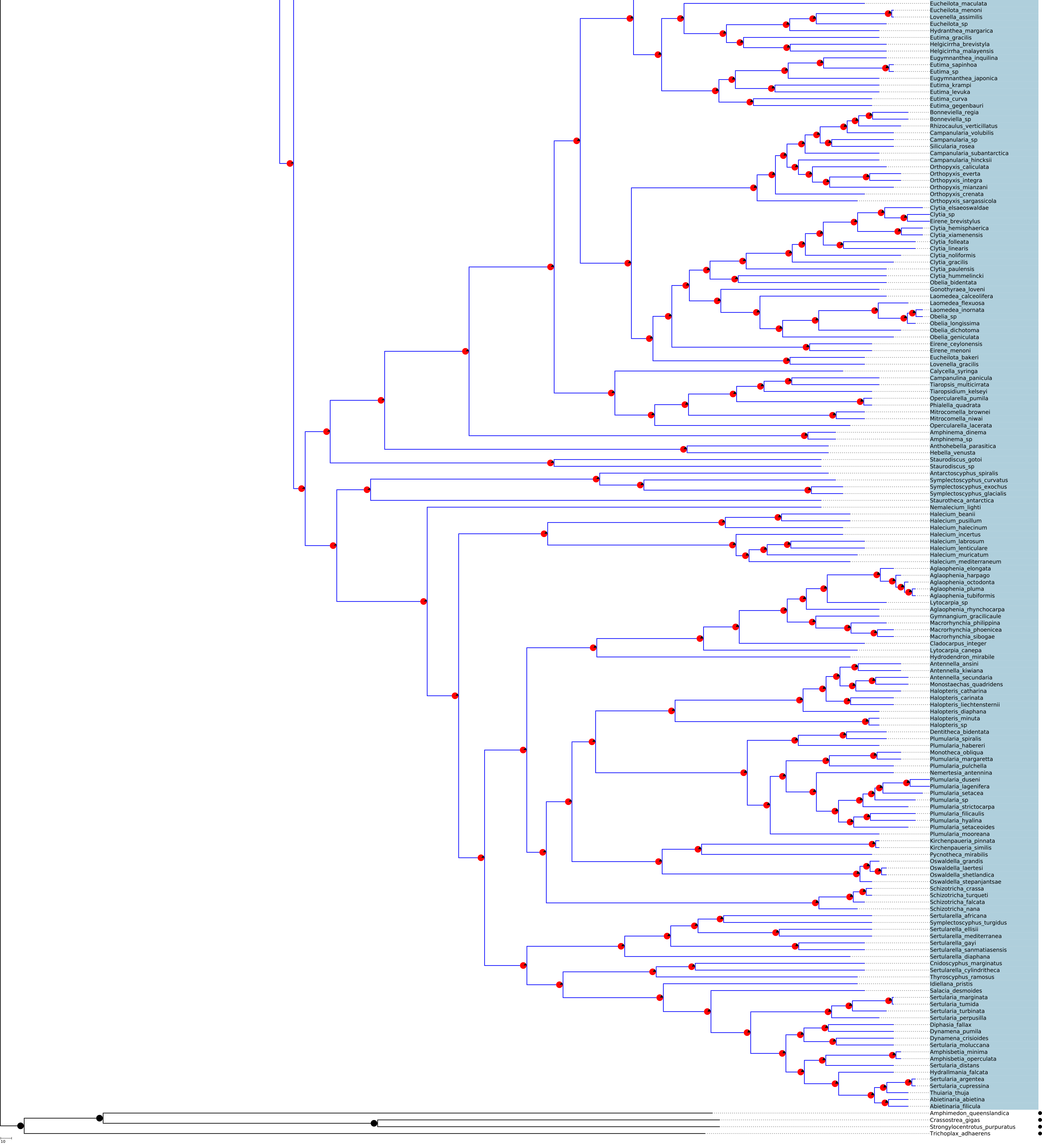
Eucheilota_maculata
Eucheilota_menoni
Lovenella_assimilis
Eucheilota_sp
Hydranthea_margarica
Eutima_gracilis
Helgicirrha_brevistyla
Helgicirrha_malayensis
Eugymnanthea_inquilina
Eutima_sapinhoa
Eutima_sp
Eugymnanthea_japonica
Eutima_krampi
Eutima_levuka
Eutima_curva
Eutima_gegenbauri
Bonneviella_regia
Bonneviella_sp
Rhizocaulus_verticillatus
Campanularia_volubilis
Campanularia_sp
Silicularia_rosea
Campanularia_subantarctica
Campanularia_hincksii
Orthopyxis_caliculata
Orthopyxis_everta
Orthopyxis_integra
Orthopyxis_mianzani
Orthopyxis_crenata
Orthopyxis_sargassicola
Clytia_elsaeoswaldae
Clytia_sp
Eirene_brevistylus
Clytia_hemisphaerica
Clytia_xiamenensis
Clytia_folleata
Clytia_linearis
Clytia_noliformis
Clytia_gracilis
Clytia_paulensis
Clytia_hummelincki
Obelia_bidentata
Gonothyraea_loveni
Laomedea_calceolifera
Laomedea_flexuosa
Laomedea_inornata
Obelia_sp
Obelia_longissima
Obelia_dichotoma
Obelia_geniculata
Eirene_ceylonensis
Eirene_menoni
Eucheilota_bakeri
Lovenella_gracilis
Calycella_syringa
Campanulina_panicula
Tiaropsis_multicirrata
Tiaropsidium_kelseyi
Opercularella_pumila
Phialella_quadrata
Mitrocomella_brownei
Mitrocomella_niwai
Opercularella_lacerata
Amphinema_dinema
Amphinema_sp
Anthohebella_parasitica
Hebella_venusta
Staurodiscus_gotoi
Staurodiscus_sp
Antarctoscyphus_spiralis
Symplectoscyphus_curvatus
Symplectoscyphus_exochus
Symplectoscyphus_glacialis
Staurotheca_antarctica
Nemalecium_lighti
Halecium_beanii
Halecium_pusillum
Halecium_halecinum
Halecium_incertus
Halecium_labrosum
Halecium_lenticulare
Halecium_muricatum
Halecium_mediterraneum
Aglaophenia_elongata
Aglaophenia_harpago
Aglaophenia_octodonta
Aglaophenia_pluma
Aglaophenia_tubiformis
Lytocarpia_sp
Aglaophenia_rhynchocarpa
Gymnangium_gracilicaule
Macrorhynchia_philippina
Macrorhynchia_phoenicea
Macrorhynchia_sibogae
Cladocarpus_integer
Lytocarpia_canepa
Hydrodendron_mirabile
Antennella_ansini
Antennella_kiwiana
Antennella_secundaria
Monostaechas_quadridens
Halopteris_catharina
Halopteris_carinata
Halopteris_liechtensternii
Halopteris_diaphana
Halopteris_minuta
Halopteris_sp
Dentitheca_bidentata
Plumularia_spiralis
Plumularia_habereri
Monotheca_obliqua
Plumularia_margaretta
Plumularia_pulchella
Nemertesia_antennina
Plumularia_duseni
Plumularia_lagenifera
Plumularia_setacea
Plumularia_sp
Plumularia_strictocarpa
Plumularia_filicaulis
Plumularia_hyalina
Plumularia_setaceoides
Plumularia_mooreana
Kirchenpaueria_pinnata
Kirchenpaueria_similis
Pycnotheca_mirabilis
Oswaldella_grandis
Oswaldella_laertesi
Oswaldella_shetlandica
Oswaldella_stepanjantsae
Schizotricha_crassa
Schizotricha_turqueti
Schizotricha_falcata
Schizotricha_nana
Sertularella_africana
Symplectoscyphus_turgidus
Sertularella_ellisii
Sertularella_mediterranea
Sertularella_gayi
Sertularella_sanmatiasensis
Sertularella_diaphana
Cnidoscyphus_marginatus
Sertularella_cylindritheca
Thyroscyphus_ramosus
Idiellana_pristis
Salacia_desmoides
Sertularia_marginata
Sertularia_tumida
Sertularia_turbinata
Sertularia_perpusilla
Diphasia_fallax
Dynamena_pumila
Dynamena_crisioides
Sertularia_moluccana
Amphisbetia_minima
Amphisbetia_operculata
Sertularia_distans
Hydrallmania_falcata
Sertularia_argentea
Sertularia_cupressina
Thuiaria_thuja
Abietinaria_abietina
Abietinaria_filicula
Amphimedon_queenslandica
Crassostrea_gigas
Strongylocentrotus_purpuratus
Trichoplax_adhaerens
10
